# The Action of Belladonna upon the Heart and Arteries, and upon the Heart in Pericarditis and Endocarditis

**Published:** 1873-01

**Authors:** David Wooster


### THE ACTION OF BELLADONNA UPON THE HEART AND ARTERIES, AND UPON THE HEART IN PERICARDITIS AND ENDOCARDITIS.

BY DAVID WOOSTER, M.D.

1. Belladonna is not a hypnotic in any just sense. It does not induce sleep by allaying general nervous irritability, nor by relieving local pain, as a neuralgia, or the pain of a pleurisy, or of a sprain, or of a phlegmon.

2. Belladonna is an excitant in small doses. For example, if five drops of the fluid extract be administered, and two hours later five drops more, in thirty minutes, or less, after the second dose, the pulse will have increased in frequency and tension, but diminished in volume. The temperature will have ascended some part of a degree, and sometimes as high as two degrees. The heart will be found to beat with increased emphasis as well as frequency, and if its rhythm was faulty before the doses, this will be found to have improved or become quite regular, the patient will experience a sensation of increased temperature, and perhaps he will find some difficulty in reading a printed page; the letters will appear to "run into each other." This dose will increase the renal excretion, and will not retard peristaltic action of the alimentary canal, but will rather add to this action.

3. Belladonna, in small doses, doubtless diminishes the caliber of the capillaries, and thus relieves peripheral congestion, and perhaps retards or suspends early inflammatory action. Large doses again, in children, will produce a genuine scarlatina of the whole body, attended with delirium and great fear, and often with a sense of choking. Under such doses the child will complain of headache and dizziness, the latter doubtless caused by aberration of vision from dilated irides. Small doses will just as surely produce paleness of the extremities in health, and diminished redness of inflamed surfaces in disease. These two effects are paradoxical in appearance rather than in reality. The drug first stimulates the muscular coat of the vessels to contraction, and this excitation being continued, the sensory nerve filaments at length lose their irritability; that is, fail to appreciate the presence of the drug; and so the vessels not only do not continue to contract, but actually dilate, in consequence of exhausted nerve irritability; and when the drug has been continued to this degree, we have congestion first of the venous and next of the arterial capillaries, and lastly of the whole circulating system; and if the drug be still continued, total anesthesia follows, and then universal paralysis, in which each individual organ participates, and the sum total is death.

4. But if belladonna is an excitant to the heart and arteries, and it is so admitted to be, and if it has the same effect applied locally as given internally, and this is admitted, then how can it be recommended in pericarditis and endocarditis? Is it not almost certain that the good effects derived from its employment must be attributed to the remedy in combination with which it has been employed? As spread on blisters applied over the precordia or the joints, given internally with aconite or digitalis, which latter are undoubted cardiac sedatives, and do undoudtedly diminish the frequency and emphasis of the heart beats, and the force, frequency and volume of the pulse?

It is exceedingly difficult to reconcile the known primary excitant action of belladonna with any antiphlogistic action in the heart affections named above, and the only hope we could have of any beneficial action from it would be in its slight diuretic effect. But this latter would by no means compensate for the nervous restlessness, increased heat, accelerated circulation, vague uneasiness and postponed sleep induced by atropia.

The only conditions of the heart in which atropia would seem to me a probable remedy would be in fatty metamorphosis or in the cardiac debility attending low fevers. It might also be of service in the exhausted condition of the heart in the last hours of endocarditis, when, to hold out any hope of life, cardiac innervation must be prolonged until the amorphous endocardial exudation shall have been so far re-absorbed as to allow the presence of the blood itself to be appreciated by the sensory filaments of the endocardial lining.—*Pacific Medical and Surgical Journal.*

